# Machine Learning Integrating ^99m^Tc Sestamibi SPECT/CT and Radiomics Data Achieves Optimal Characterization of Renal Oncocytic Tumors

**DOI:** 10.3390/cancers15143553

**Published:** 2023-07-09

**Authors:** Michail E. Klontzas, Emmanouil Koltsakis, Georgios Kalarakis, Kiril Trpkov, Thomas Papathomas, Apostolos H. Karantanas, Antonios Tzortzakakis

**Affiliations:** 1 Department of Medical Imaging, University Hospital of Heraklion, Heraklion 71110, Greece; miklontzas@gmail.com (M.E.K.); akarantanas@gmail.com (A.H.K.); 2 Computational BioMedicine Laboratory, Institute of Computer Science, Foundation for Research and Technology (FORTH), Heraklion 70013, Greece; 3 Department of Radiology, School of Medicine, University of Crete, Voutes Campus, Heraklion 71110, Greece; 4 Department of Diagnostic Radiology, Karolinska University Hospital, Stockholm 17177, Sweden; emmanouil.koltsakis@regionstockholm.se (E.K.); georgios.kalarakis@regionstockholm.se (G.K.); 5 Division of Radiology, Department for Clinical Science, Intervention and Technology (CLINTEC), Karolinska Institutet, Stockholm 14152, Sweden; 6 Alberta Precision Labs, Department of Pathology and Laboratory Medicine, Cumming School of Medicine, University of Calgary, Calgary, AB T2L 2K5, Canada; kiril.trpkov@albertaprecisionlabs.ca; 7 Institute of Metabolism and Systems Research, University of Birmingham, Birmingham B15 2TT, UK; thomas.papathomas@vestreviken.no; 8 Department of Clinical Pathology, Vestre Viken Hospital Trust, Drammen 3004, Norway; 9 Medical Radiation Physics and Nuclear Medicine, Section for Nuclear Medicine, Karolinska University Hospital, Huddinge, Stockholm 14186, Sweden

**Keywords:** ^99m^Tc Sestamibi SPECT/CT, artificial intelligence, machine learning, radiomics, renal cell carcinoma, renal oncocytoma, renal oncocytic neoplasia, XGboost

## Abstract

**Simple Summary:**

This study focuses on the integration of ^99m^Tc Sestamibi SPECT/CT and radiomics analysis to characterize benign renal oncocytic neoplasia. Our research includes renal tumors with histopathological analysis (conducted by independent pathologists) serving as the ground truth. Radiomics data were extracted from contrast-enhanced CT images to build machine-learning models. The combined SPECT/radiomics model achieved higher accuracy (95%) than the radiomics-only model (75%) and visual evaluation of ^99m^Tc Sestamibi SPECT/CT alone (90.8%). This approach promises the improvement of diagnostic accuracy in renal tumor characterization and the reduction in unnecessary surgery for benign tumors.

**Abstract:**

The increasing evidence of oncocytic renal tumors positive in ^99m^Tc Sestamibi Single Photon Emission Tomography/Computed Tomography (SPECT/CT) examination calls for the development of diagnostic tools to differentiate these tumors from more aggressive forms. This study combined radiomics analysis with the uptake of ^99m^Tc Sestamibi on SPECT/CT to differentiate benign renal oncocytic neoplasms from renal cell carcinoma. A total of 57 renal tumors were prospectively collected. Histopathological analysis and radiomics data extraction were performed. XGBoost classifiers were trained using the radiomics features alone and combined with the results from the visual evaluation of ^99m^Tc Sestamibi SPECT/CT examination. The combined SPECT/radiomics model achieved higher accuracy (95%) with an area under the curve (AUC) of 98.3% (95% CI 93.7–100%) than the radiomics-only model (71.67%) with an AUC of 75% (95% CI 49.7–100%) and visual evaluation of ^99m^Tc Sestamibi SPECT/CT alone (90.8%) with an AUC of 90.8% (95%CI 82.5–99.1%). The positive predictive values of SPECT/radiomics, radiomics-only, and ^99m^Tc Sestamibi SPECT/CT-only models were 100%, 85.71%, and 85%, respectively, whereas the negative predictive values were 85.71%, 55.56%, and 94.6%, respectively. Feature importance analysis revealed that ^99m^Tc Sestamibi uptake was the most influential attribute in the combined model. This study highlights the potential of combining radiomics analysis with ^99m^Tc Sestamibi SPECT/CT to improve the preoperative characterization of benign renal oncocytic neoplasms. The proposed SPECT/radiomics classifier outperformed the visual evaluation of ^99m^Tc Sestamibii SPECT/CT and the radiomics-only model, demonstrating that the integration of ^99m^Tc Sestamibi SPECT/CT and radiomics data provides improved diagnostic performance, with minimal false positive and false negative results.

## 1. Introduction

Accumulating evidence exists nowadays that indolent renal tumors appear positive in ^99m^Tc Sestamibi Single Photon Emission Tomography/Computed Tomography (SPECT/CT) [1,2,3,4]. Detecting clinically benign renal tumors using ^99m^Tc-Sestamibi SPECT/CT could safely reduce the overtreatment of those tumors. Compared to traditional CT, 99mTc-Sestamibi SPECT/CT is a hybrid imaging method that combines the depiction 99mTc-Sestamibi with CT image acquisition. The broad spectrum of renal oncocytic tumors is reflected in the latest update of the 5th World Health Organization (WHO) edition of kidney tumor pathology [5,6]. Apart from the well-studied tumors like renal oncocytoma (RO) and chromophobe renal cell carcinoma (chRCC), new entities like hybrid oncocytic chromophobe tumors (HOCT) [7] and low-grade oncocytic tumors (LOT) [8] are getting established via modern histopathologic, immunohistochemical, and molecular methods and clinical evidence. The establishment of new and emerging renal entities [9] requires, however, an understanding of the diagnostic nuances of clinical oncologists and pathologists [10] in differentiating such renal oncocytic neoplasia. According to the latest guidelines from the WHO, the new and emerging renal entities impose an updated approach to the diagnostic performance of this examination method, namely ^99m^Tc-Sestamibi SPECT/CT.

Previous research of our group has uncovered metabolic differences between tumors positive on ^99m^Tc Sestamibi SPECT/CT, contributing to the expansion of the renal oncocytic neoplasia [11]. Renal tumors positive on ^99m^Tc Sestamibi SPECT/CT examination are potentially benign (RO and LOT) [12,13] or indolent (HOCT) [2] with no proven metastatic potential. The high negative predictive value (~90%) of this hybrid imaging method, can reduce the overtreatment of low-grade oncocytic neoplasia [14] by proposing a cost-effective oncological investigation model of renal neoplasia [15]. Kidney tumors negative on ^99m^Tc Sestamibi SPECT/CT examination could be managed with surgery. In contrast, those that are positive could be followed by an active surveillance program [16], avoiding unnecessary surgery [17]. 

The preoperative differentiation of benign renal oncocytic neoplasms, chRCC, and other RCC types remains a diagnostic challenge for conventional radiological methods [18]. Modern radiomics advances [19] and the implementation of artificial intelligence models [20] contribute to the non-invasive characterization of renal neoplasia but not in a definitive way. The latest study of our group [2] visually evaluated the performance of ^99m^Tc Sestamibi SPECT/CT examination in 57 renal tumors resulting in sensitivity and specificity of 88% and 85%, respectively, in the characterization of RO and HOCT when clustered together. Further, an increase in sensitivity and specificity of this method would further reduce the false positive and false negative findings, increasing the method’s reliability and ultimately avoiding unnecessary surgery for such benign tumors.

Radiomics analysis provides a detailed evaluation of the regions of interest in medical images, allowing for the quantification and analysis of image characteristics imperceptible to the experienced human eye. It represents the image-based equivalent of other traditional -omics methods (e.g., metabolomics, transcriptomics, genomics, proteomics, etc.) and has the potential to offer an image-based characterization of complex tissues. Areas of interest in medical images, such as CT or MRI, are segmented, and high-dimensional data are extracted to provide a high-fidelity analysis of the examined tissues. This includes a series of “semantic” and “agnostic” features. Semantic features such as the size and shape of a lesion are features common in radiology reports, whereas agnostic features include texture details that attempt to quantify tumor heterogeneity [21]. This strategy could be used as an image-based biopsy for tumors [22], alone or in combination with other biological tumor traits such as the uptake of ^99m^Tc Sestamibi. Many radiomics studies have been published attempting to differentiate between benign and malignant renal tumors. Nonetheless, most were based on prior WHO classifications of renal tumors, thus neglecting newly recognized tumor types with different biologic characteristics. In addition, most reported unacceptably high false positive and false negative findings that hindered their introduction into the clinical practice [23]. Thus, identifying strategies to increase the diagnostic accuracy of radiomics would be an important addition to existing radiomics models.

This study attempted to combine the diagnostic performance of radiomics analysis and ^99m^Tc Sestamibi SPECT/CT in differentiating RO, HOCT, and LOT from RCC. The aim was to increase the specificity of existing radiomics methods by integrating 99mTc Sestamibi uptake information while utilizing an up-to-date version of renal tumor classification. This combined SPECT/radiomics model was compared to a model based only on radiomics and the one that utilized visual ^99m^Tc Sestamibi SPECT/CT evaluation for the characterization of benign and indolent renal oncocytic neoplasia.

## 2. Materials and Methods

The research project was conducted at the Nuclear Medicine Department of Karolinska University Hospital, Huddinge, following ethical approval from the Regional Ethical Review Board and the local Radiation Safety Committee. Written informed consent was obtained from all participants in this study. This study spanned from September 2015 to September 2019 and involved a non-randomized, exploratory research design. Eligible candidates were discussed at the kidney tumor multidisciplinary conference in the hospital’s Radiology Department, excluding patients with advanced-stage renal tumors or metastatic disease.

### 2.1. Patients and ^99m^Tc Sestamibi SPECT/CT Imaging

A total of 57 consecutive renal tumors from 52 patients were prospectively collected as part of the MIDOR Project (Swedish innovation agency, VINNOVA), as published previously by our group [2]. Following a standardized imaging protocol [24], participants who agreed to participate underwent a ^99m^Tc-Sestamibi SPECT/CT examination before nephrectomy or renal biopsy. The visual evaluation of ^99m^Tc-Sestamibi uptake was performed independently by two experienced readers for all 57 tumors [2]. The uptake was categorized as Sestamibi-positive if it visually exceeded the uptake in the normal renal parenchyma and as Sestamibi-negative if it was equal to or lower than the uptake in the normal parenchyma [2]. A priori sample size estimation was performed using a linear regression model type, significance set at 0.05, an average of 10 model predictors, power of 0.8, and an effect size of 0.3, which yielded a minimum sample size of 48 samples. 

### 2.2. Pathological Diagnosis

Two independent pathologists conducted the histopathological analysis of the examined renal tumors. They reviewed hematoxylin and eosin (H&E) and immunohistochemical (IHC) slides of the tumors in a blinded manner, without knowledge of any imaging evaluation. The confirmed or updated histopathological diagnoses, determined via consensus, were used as the ground truth for correlation with the radiomics and SPECT/CT results [2]. Furthermore, a third expert urologic pathologist blindly evaluated all chRCC included in the MIDOR study that evaluated the metabolic differences between Sestamibi-positive and Sestamibi-negative chRCCs [2,11]. The correlation of the histopathologic analysis with metabolomics and SPECT/CT results resulted in the inclusion of a renal tumor group of LOT, an emerging benign entity. The tumors in this study have been grouped based on this contemporary diagnostic evaluation, in which HOCT and LOT are considered in the same group as RO.

### 2.3. CT-Based Radiomics Analysis

Contrast-enhanced CT examinations in the venous phase of 54 of 57 renal tumors were retrieved to extract radiomics data. Due to insufficient CT material (lack of venous phase images), 3 of 57 tumor cases were not included in the radiomics analysis. Each tumor was manually segmented by two experienced readers in image segmentation using 3D Slicer v. 4.11.20 for Windows (Slicer.org (accessed on 8 July 2023)); radiomics data were extracted using the PyRadiomics module of 3DSlicer. Original and texture features (grey level co-occurrence matrix, grey level dependence matrix, grey level run length matrix, grey level size zone matrix, neighboring grey tone difference matrix), and their wavelet and Laplacian of Gaussian transformations were also extracted. Voxels were resized to 1 × 1 × 4 mm, and a uniform bin width of 32 was used to homogenize the image information. A total of 944 features were extracted from each volume of interest. Radiomics feature stability during segmentation was assessed, and only features with a coefficient of variation <10% between duplicate segmentations were utilized, leading to a final dataset of 700 features. Data were randomly split 70%/30% into training and validation sets. Each set was subjected to scaling, elimination of correlated features, and Boruta tree-based feature selection.

### 2.4. Machine Learning

**Figure 1:**
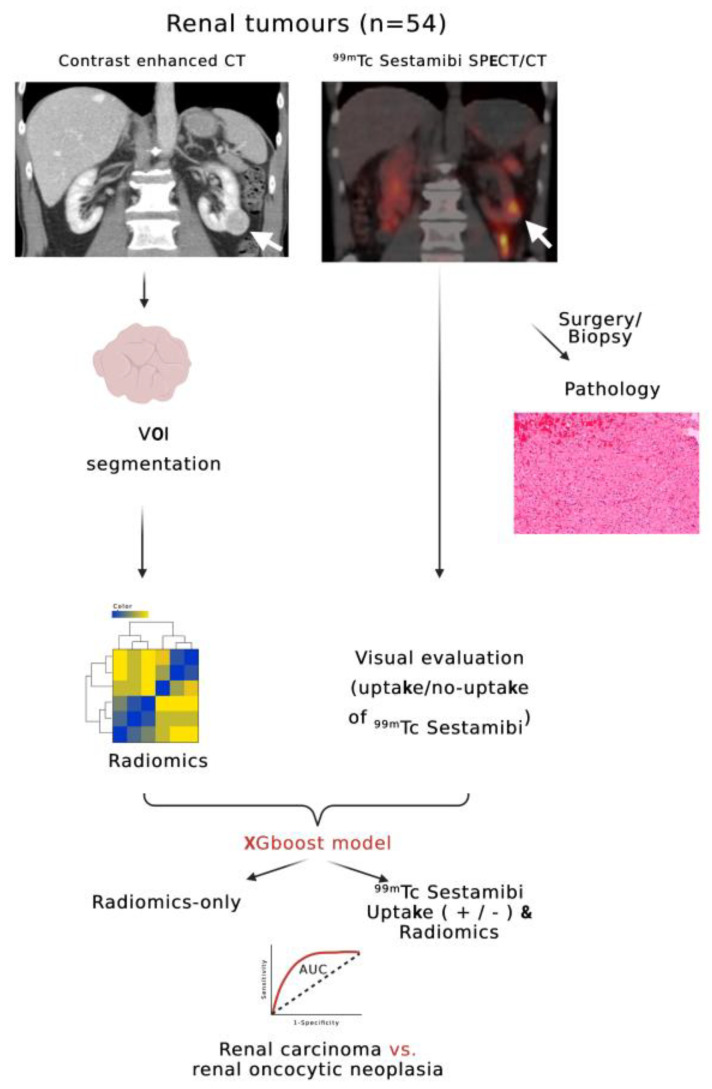
Flowchart outlining the design of the radiomics arm of this study. White arrows indicate a renal tumor of the inferior pole of the left kidney with visible ^99m^Tc Sestamibi SPECT/CT uptake (Sestamibi-positive) (created with biorender.com, accessed on 12 June 2023).

Features identified as significant by Boruta were used for XGBoost classifier construction. Two XGboost models were trained: (a) one only with radiomics features and (b) one with the addition of ^99m^Tc Sestamibi uptake as an extra model feature (referred to further as SPECT/radiomics). XGboost was chosen based on its superior performance on tabular data, which validated it as one of the most successful machine-learning models in data science competitions using tabular data (https://github.com/dmlc/xgboost/blob/master/demo/README.md#machine-learning-challenge-winning-solutions, accessed on 12 June 2023). XGboost combines a set of weak learners (decision trees) to construct a powerful classifier. Details about the structure of XGboost and relevant code for the replication of the model can be found in the documentation of the R package (https://xgboost.readthedocs.io/en/stable/R-package/index.html, accessed on 2 July 2023). The model can also be used in python with the xgboost package. Models were trained with 5-fold cross-validation in the training set and hyperparameter optimization with random search (n = 1000). Classifier performance was evaluated with areas under the curve (AUC) of receiver operating characteristics (ROC) curves and the respective 95% confidence intervals. Confidence intervals were calculated via bootstrapping (n = 10,000). Optimal probability thresholds were defined from ROC curves to maximize sensitivity, specificity, accuracy, F1-scores, positive predictive values (PPV), and negative predictive values (NPV). AUC of different ROC curves were statistically compared using bootstrapping. Weight importance of features for XGboost classification was analyzed and presented on feature importance graphs. Weight importance quantifies the contribution of each feature to the final classification based on the number of times it was used to split the data on the ensemble trees composing the XGboost model. The more frequently a feature is used for splitting, the higher its weight importance. Significance was considered with *p*-values less than 0.05. Model training was performed with R programming language (v 4.2.2 as implemented in R Studio v2023.06 for Mac), using an Apple M1 Max 64 Gb system with MacOs Ventura 13.3.1. The outline of the radiomics arm of our study design is illustrated in Figure 1.

## 3. Results

### 3.1. Tumor Characteristics in ^99m^Tc Sestamibi SPECT/CT

The visual evaluation of ^99m^Tc-Sestamibi SPECT/CT examinations was performed for the whole dataset of 57 tumors. A total of 19 tumors were included in the benign oncocytic group that contained 11 RO, 5 HOCT, and 3 LOT. When clustering RO, HOCT, and LOT, the method yielded a sensitivity of 89.5% and a specificity of 92.1%. Only 2 of 19 tumors in the benign category were negative for ^99m^Tc Sestamibi. In contrast, the majority of other tumors were negative for ^99m^Tc Sestamibi, with only three chromophobe RCCs displaying uptake. The negative predictive value of this hybrid molecular method reached 94.6%, with a positive predictive value of 85%, Table 1.

### 3.2. Radiomics Analysis

**Figure 2:**
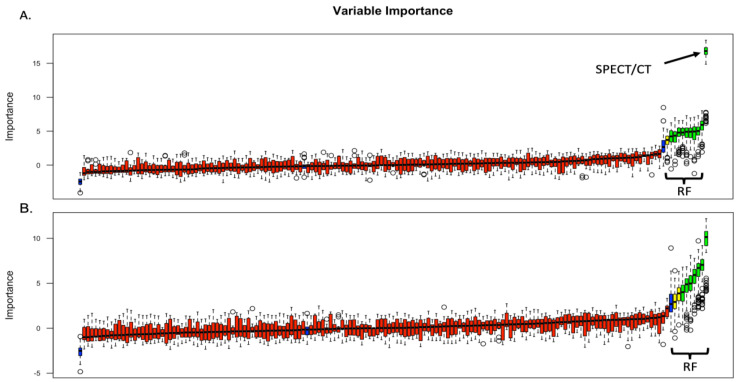
Boruta feature selection results in the SPECT/radiomics (**A**) and radiomics-only (**B**) groups. RF: radiomics features; SPECT/CT: uptake (±) of 99mTc Sestamibi on SPECT/CT. Blue box, red, yeallow and green plots represent shadow, not important, tentative and confirmed important attributes, respectively.

Radiomics data were extracted for the 54 tumors where venous phase images were available. Two groups were evaluated: (a) group only with radiomics data and (b) group where ^99m^Tc Sestamibi SPECT/CT uptake was included together with the radiomics features (SPECT/radiomics group). Following collinearity correction, Boruta feature selection yielded 12 significant features in the SPECT/radiomics group, including the uptake of ^99m^Tc Sestamibi (Figure 2A). In the radiomics-only group, Boruta identified 10 significant radiomics features used for subsequent model building (Figure 2B).

**Figure 3:**
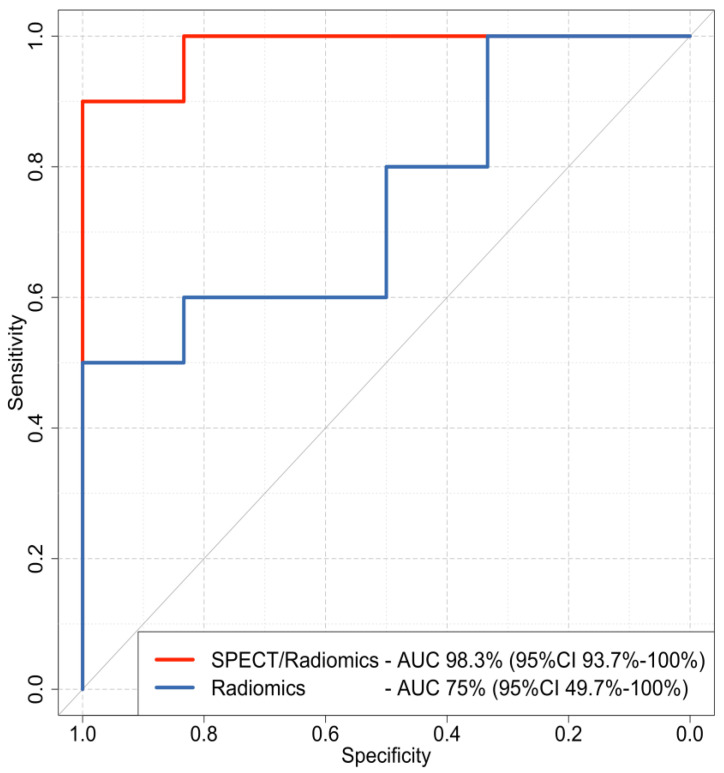
Receiver operating characteristics (ROC) curves of the combined SPECT/radiomics (red line) and the radiomics-only (blue line) XGboost models. AUC: area under the curve; CI: confidence interval.

The features identified by Boruta were then used to build two XGboost classifiers. The combined SPECT/radiomics classifier achieved an AUC of 98.3 (95%CI 93.7% to 100%), which was significantly higher (*p* = 0.048) than the AUC of the radiomics-only model with an AUC of 75% (95%CI 49.7% to 100%) (Figure 3). The combined SPECT/radiomics method also demonstrated higher sensitivity and specificity than the visual evaluation of ^99m^Tc Sestamibi SPECT/CT alone. However, the NPV of the SPECT-only method was still higher than the respective NPV of the combined SPECT/radiomics classifier (94.6% vs. 85.71%, respectively). Detailed performance metrics for all methods are presented in Table 2.

**Figure 4:**
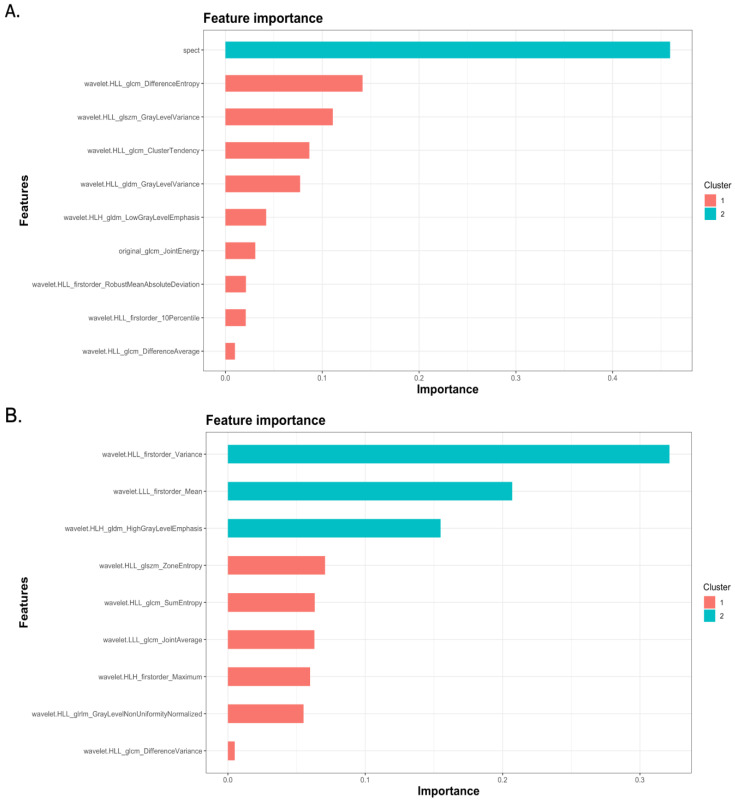
Feature important graphs of XGboost models demonstrating features important for the performance of the combined SPECT/radiomics (**A**) and the radiomics-only (**B**) model.

**Figure 5:**
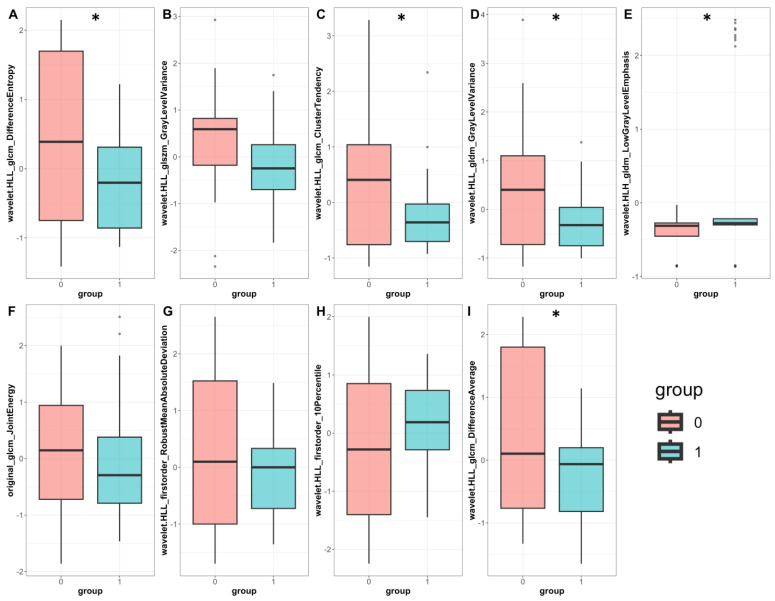
Individual radiomics feature values were important for classifying the combined SPECT/radiomics model. Barplots represent normalized feature values in the benign (pink) and malignant (cyan); six wavelet transformations of texture features (**A**–**E**,**I**), two wavelet transformations of first-order features (**G**,**H**), and one original texture radiomics feature (**F**) are compared between the benign and malignant group. Boxplot statistics have been derived with the geom_boxplot function of the ggplot2 package in R; y-axis: standardized relative value of radiomics features; dots represent outlier data beyond the end of the whiskers *: *p* < 0.05.

Feature importance analysis demonstrated that ^99m^Tc Sestamibi uptake was the most important attribute that influenced the prediction of the combined model, along with eight wavelets and one original radiomics feature (Figure 4A). In the radiomics-only model, seven wavelet transformations of texture features and two wavelet transformations of original features were found to determine the performance of the model (Figure 4B). Individual values of important radiomics features from the combined SPECT/radiomics model can be found in Figure 5. As shown in Figure 5, the values of only five of nine important features differed significantly between these two groups, highlighting the need for inclusive multivariate models where interactions and simultaneous level changes of the features can be important for the final prediction.

## 4. Discussion

In this study, we combined the radiomics analysis with ^99m^Tc Sestamibi SPECT/CT to optimize the preoperative differentiation between malignant renal tumors and a group of benign oncocytic neoplasms. A combined SPECT/radiomics machine-learning classifier was developed, which achieved better performance compared to the radiomics-only classifier and the exclusive visual evaluation using ^99m^Tc Sestamibi SPECT/CT. Importantly, we used a contemporary approach in diagnosing oncocytic renal tumors, that included LOT, as a recently recognized benign entity.

Many radiomics studies have been published attempting to differentiate between benign and malignant renal tumors. A systematic review of such studies identified 52 publications until 2021 which attempted such a differentiation, highlighting great heterogeneity between radiomics methodologies and the overall low quality of the studies [23]. Published CT-based radiomics studies reported AUCs between 67% and 93% [25,26,27,28,29,30] and utilized a variety of machine-learning models, including regression models [31], random forests [28], and gradient-boosting algorithms [29], using histopathology as the ground truth [20]. The study by Deng et al. [32], which utilized one of the largest cohorts of such patients (n = 501), demonstrated an AUC of ~62% for the differentiation between benign and malignant tumors based on texture features. Our results are comparable to those published in the literature, indicating that radiomics alone can distinguish benign oncocytic neoplasia with an AUC of ~75%. This performance was, however, significantly increased by combining the ^99m^Tc Sestamibi uptake as an additional parameter, resulting in increased diagnostic accuracy and improved positive and negative predictive value over the radiomics-only approach. The addition of non-radiomics parameters to a multivariable model has been previously suggested by Lambin et al. [33], as a method to optimize the predictive model development. We found that the uptake of ^99m^Tc Sestamibi distinguished the metabolic behavior of the renal tumors and discriminated specifically a group of benign oncocytic neoplasms. Consequently, the addition of ^99m^Tc Sestamibi SPECT/CT has been recently incorporated for the evaluation of renal tumors in the 2023 version of the Swedish national guidelines for the management of renal cancer (https://cancercentrum.se/samverkan/cancerdiagnoser/njure/vardprogram/, accessed on 12 June 2023). Therefore, integrating ^99m^Tc-Sestamibi SPECT/CT for the preoperative clinical examination could guide clinical decisions toward active surveillance or surgical intervention.

Combined SPECT/radiomics methodology achieved better sensitivity and specificity than the visual evaluation of ^99m^Tc Sestamibi uptake. This is important because such a combined method can reduce the false positive and the false negative findings, which may lead to unnecessary surgery delays or increase unnecessary surgeries, affecting patient morbidity and mortality. Nonetheless, the NPV of ^99m^Tc-Sestamibi SPECT/CT alone was higher than the NPV of the combined method. This could be attributed to the fact that radiomics features alone bear a very low NPV (~55%), which dilutes the excellent NPV of ^99m^Tc-Sestamibi SPECT/CT visual evaluation. As demonstrated in our previous work and by other research groups, most ROs are Sestamibi-positive [14]. Therefore, a combined SPECT/radiomics model could be of clinical value in cases where ^99m^Tc-Sestamibi SPECT/CT falsely indicates signs of malignancy.

Radiomics-only and combined SPECT/radiomics models are based mainly on wavelet transformations of texture features. This is likely because the wavelet transformations of original images decompose the image using low and high-band filters, and bypass variations in noise profiles, which may introduce a degree of examination variance. In addition, wavelet transformations are known to enhance line and edge patterns on images [34], which are present, for example, on irregular surfaces, fibrotic areas, and calcifications, in renal tumors [35]. Similar wavelet features have been previously shown to be important in the radiomics analysis of areas containing irregular lines such as the band lines of avascular bone necrosis [36]. The fact that such texture features were important for the classification is also in line with the current literature, as demonstrated by Bhandari et al. in a systematic review of radiomics for renal tumor analysis [37]. The authors pointed out that texture features have been important in most of the selected studies, and they identified the need for well-designed prospective studies to assess the value of radiomics in classifying renal neoplasia. CT is adequate for the analysis of the fine structure of hard and soft materials [38,39,40], being an excellent method for the extraction of fine radiomics information. These features can be used with in silico approaches that are important in simulating conditions and data that are not readily available in research and clinical practice, reducing experimental time and associated costs [41,42,43]. The analysis of individual levels of important features showed differences between tumor groups, with some of the features reaching statistical significance. This highlights the importance of using multivariate analysis and multivariate prediction models because single feature values cannot sufficiently discriminate the tumor-imaging complexity.

This work has certain strengths and limitations. First, it includes a prospectively collected dataset with histopathologically proven ground truth, including the most recent classification of renal tumors, according to the WHO. In addition, it integrated radiomics evaluation with ^99m^Tc Sestamibi uptake as an additional parameter, resulting in a multifaceted renal tumor evaluation. The limitations include the lack of an external validation dataset and the limited sample size. However, the number of evaluated tumors is higher than the sample size required to achieve a power of 80% with a statistical significance of 0.05, determined using sample size calculation. We hope that our initial results will prompt further studies for external validation of the proposed algorithm that will confirm the method’s applicability and usefulness in routine clinical practice to reduce unnecessary surgeries for benign renal tumors.

## 5. Conclusions

This study describes a machine-learning method that combined ^99m^Tc Sestamibi SPECT/CT and radiomics data to preoperatively distinguish benign oncocytic renal neoplasms. In contrast to existing radiomics studies, we have utilized the most up-to-date pathological classification of renal tumors where benign RO is grouped with HOCT and LOT. The utilization of the ^99m^Tc Sestamibi SPECT/CT method follows the recently adopted practices introduced into clinical practice guidelines.

The results of this study demonstrate that the integration of ^99m^Tc Sestamibi SPECT/CT and radiomics data provides improved diagnostic performance, with minimal false positive and false negative results, reaching an accuracy of 95%. We hope that further external validations of our algorithm will confirm the method’s applicability in various clinical settings and will reduce the overtreatment of benign renal tumors. CT-based radiomics could also supplement ^99m^Tc Sestamibi SPECT/CT examinations for accurate preoperative characterization of renal neoplasia.

## Figures and Tables

**Table 1:** Visual evaluation of ^99m^Tc-Sestamibi uptake on 57 solid renal tumors resulting in 89.4% sensitivity and 92% specificity in detecting RO, HOCT, and LOT. Histological types in bold font represent the benign oncocytic group. HOCT: Hybrid oncocytic chromophobe tumor. LOT: Low-grade oncocytic tumor.

Histological Types of Renal Tumors	Number of Renal Tumors	^99m^Tc-Sestamibi Positive, n (%)	^99m^Tc-Sestamibi Negative, n (%)
Renal Oncocytoma	11	9 (82%)	2 (18%)
HOCT	5	5 (100%)	0
LOT	3	3 (100%)	0
Chromophobe RCC	8	3 (37.5%)	5 (62.5%)
Clear Cell RCC	13	0	13 (100%)
Papillary RCC	9	0	9 (100%)
Clear cell Papillary Renal Cell Tumor	4	0	4 (100%)
Collision RCC	1	0	1 (100%)
B-cell Lymphoma	1	0	1 (100%)
Metanephric adenoma	1	0	1 (100%)
Angiomyolipoma	1	0	1 (100%)

**Table 2:** Performance metrics various methods.

	AUC	Accuracy	F1-Score	Sensitivity	Specificity	PPV	NPV
SPECT & Radiomics	98.3% (93.7–100%)	95%	90%	90%	100%	100%	85.71%
Radiomics	75% (49.7–100%)	71.67%	70.59%	60%	83.3%	85.71%	55.56%
Visual evaluation of ^99m^Tc Sestamibi SPECT/CT	90.8% (82.5–99.1%)	90.8%	87.2%	89.5%	92.1%	85%	94.6%

## Data Availability

The data presented in this study are available on reasonable request from the corresponding author.
